# Does Being Ignored on WhatsApp Hurt? A Pilot Study on the Effect of a Newly Developed Ostracism Task for Adolescents

**DOI:** 10.3390/jcm12052056

**Published:** 2023-03-06

**Authors:** Delia Latina, Andreas Goreis, Polona Sajko, Oswald D. Kothgassner

**Affiliations:** 1 Center for Lifespan Developmental Research, Örebro University, 702 81 Örebro, Sweden; 2 Department of Child and Adolescents Psychiatry and Psychotherapy, University of Ulm, 89081 Ulm, Germany; 3 Department of Child and Adolescent Psychiatry, Medical University of Vienna, 1090 Vienna, Austria; 4 Klinik für Kinder- und Jungendpsychiatrie, Psychosomatik und Psychotherapie Christophsbab, 73035 Göppingen, Germany

**Keywords:** adolescence, depression, non-suicidal self-injury, simulated online ostracism task, heart rate, heart rate variability, emotional affect, instant-messaging-communication platforms

## Abstract

(1) Background: Many studies have used a well-known social exclusion task, namely Cyberball, to assess the psychophysiological reactions to ostracism in laboratory settings. However, this task has been recently criticized for its lack of realism. Instant messaging communication platforms are currently central communication channels where adolescents conduct their social life. These should be considered when recreating the emotional experiences that fuel the development of negative emotions. To overcome this limitation, a new ostracism task, namely SOLO (Simulated On-Line Ostracism), recreating hostile interactions (i.e., exclusion and rejection) over WhatsApp was developed. The aim of this manuscript is to compare adolescents’ self-reported negative and positive affect, as well as physiological reactivity (i.e., heat rate, HR; heart rate variability, HRV) exhibited during SOLO to Cyberball. (2) Method: A total of 35 participants (Mage = 15.16; SD = 1.48; 24 females) took part in the study. The first group (*n* = 23; transdiagnostic group), recruited at an inpatient and outpatient unit of a clinic for children and adolescent psychiatry, psychotherapy, and psychosomatic therapy in Baden-Württemberg (Germany), reported clinical diagnoses linked with emotional dysregulation (e.g., self-injury and depression). The second group (n = 12; control group), recruited in the district of Bavaria and Baden-Württemberg, had no pre-existing clinical diagnoses. (3) Results: The transdiagnostic group showed higher HR (b = 4.62, *p* < 0.05) and lower HRV (b = 10.20, *p* < 0.01) in SOLO than in Cyberball. They also reported increased negative affect (interaction b = −0.5, *p* < 0.01) after SOLO but not after Cyberball. In the control group, no differences in either HR (*p* = 0.34) or HRV (*p* = 0.08) between tasks were found. In addition, no difference in negative affect after either task (*p* = 0.83) was found. (4) Conclusion: SOLO could be an ecologically valid alternative to Cyberball when assessing reactions to ostracism in adolescents with emotional dysregulation.

## 1. Introduction

The peer context, in general, and adverse peer experiences, in particular, seem to have strong implications on emotional development during adolescence [1]. In this phase of life, adolescents look at peers for a sense of inclusion, support, and security [2]. Thus, acceptance from peers represents a critical aspect of positive emotional development [3,4,5]. In addition, adolescence is well known for its higher sensitivity to peer evaluation and feedback [6,7]. In this scenario, experiences of ostracism in the form of social exclusion and peer rejection seem to influence the physical and psychological wellbeing of adolescents [8,9,10]. Overall, negative experiences, such as ostracism, can serve as fuel for the development of those negative emotions that are particularly difficult to regulate.

Although the experience of peers’ ostracism could be maladaptive for all adolescents, it could be particularly problematic for those that are more vulnerable. For example, a high percentage of adolescents who report depressive symptoms [11] or non-suicidal self-injury (hereafter only “self-injury”) [12] have been excluded or rejected by their peers. These groups of adolescents are characterized by a high inability to regulate their negative emotions in an adaptive manner [13,14]. Different models can help explain why these adolescents are particularly vulnerable to peers’ ostracism. On the one hand, the cognitive model of depression [15,16] postulated that adolescents who suffer from depression have automatic and uncontrollable negative thoughts about themselves (“I’m worthless”), their environment (“Nobody loves me”), as well as their future (“I will never have any success in life”). These negative schemas can be activated by stressful events or by a chain of events (e.g., ostracism), and they tend to skew the information processing system, which directs the attention of the adolescent toward negative stimuli. This translates a specific neutral experience into a negative interpretation, which tends to exacerbate depressive symptoms. On the other hand, the functional model of non-suicidal self-injury [17,18] or the experiential avoidance model of self-injury (EAM) [19] conceptualized self-injury as an emotional regulation strategy, which is used to regulate negative affect. Studies report how these negative emotions mostly occur during stressful interactions with peers and interactions that include several forms of ostracism [20,21], and how they tend to decrease right after the use of self-injury [17]. Overall, the current literature seems to point to peers’ ostracism as an emotionally upsetting experience that can have a major impact on adolescents’ psychological wellbeing, especially for those adolescents who have difficulties regulating negative emotions in an adaptive way.

To better understand how ostracism is linked to the emotional and, therefore, psychosocial wellbeing of adolescents, scholars have tried to replicate these socially upsetting experiences in laboratory settings. More specifically, they assessed the physiological stress effects of ostracism on different parameters of the autonomic nervous system (ANS), including heart rate (HR) and heart rate variability (HRV) [22,23,24]. Through the interplay between the sympathetic and parasympathetic systems, the ANS has the objective to respond to changes in environmental conditions in an adaptive manner and is therefore considered a crucial node in the relation between psyche and soma. According to the neurovisceral integration model [25,26], a dysregulation of the ANS is linked to emotional dysregulation. In this respect, several studies showed lower HRV in conditions of deficient emotional regulation, including depression [27,28], as well as difficulties in controlling impulsive behaviors (e.g., self-injury) when experiencing negative emotions in daily life [29].

To simulate ostracism that showed reliable effects in eliciting negative affect, Williams and colleagues [30] introduced the Cyberball task with the aim of assessing the psychophysiological reactions to social exclusion and peer rejection in social psychological experiments. Cyberball is a computerized virtual ball-tossing game played between the participant and other virtual players [30]. Specifically, the participant is invited to play an online ball-tossing game, which they believe to be playing with two other people currently sitting elsewhere. However, the other two participants are simply standardized computer players. The entire game is then divided into two sections: an “inclusion” section, during which all the players receive and toss the ball equally, and an “exclusion” section, where the other two members (computer players) toss the ball to each other, excluding the actual participant, and thus eliciting feelings of social exclusion. Cyberball sets itself apart from other paradigms tailored to study rejection [31,32,33] in that participants are not explicitly informed about their exclusion. This manipulation allows the study of the primary reaction to being ignored and excluded by peers.

To the best of our knowledge, since 2000, hundreds of studies have used the Cyberball task to assess the psychophysiological reactions to ostracism in social psychological experiments both in adults and adolescents [24,34,35]. These studies have reported higher negative affect and lower positive mood [23,36,37], as well as an increase in heart rate and altered cortisol levels [22,23] after exposure to the task. This also seems to be the case for adolescents reporting depression and non-suicidal self-injury [38,39]. Despite its broad use, this task has been recently criticized for its lack of realism [40] and, thus, ecological validity. For example, although Cyberball allows for the inclusion of pictures above each figurine to represent a participant, the abstract stimulus bears only minimal resemblance to the social interactions that people have daily. To confirm this critique, scholars reported that the social pain followed by exposure to Cyberball is less intense compared to that following a more personal form of social rejection [41,42]. To overcome this limitation, researchers have made use of virtual reality and developed a virtual counterpart of Cyberball. By creating a sense of “presence” within the situation, this new paradigm should be able to evoke reactions that are comparable to real-life situations. Some studies have reported preliminary results on the usefulness of this new virtual reality version of Cyberball [22,43,44]. Despite virtual reality making Cyberball approximate real-life settings, it still does not fully reflect real-life situations that people experience daily.

Since the introduction of smartphones, people belonging to “Generation Z” (born between 1995 and 2012) are the first ones to become immersed in technologies with the possibility of creating their own media content [45,46]. In the last few years, instant messaging communication platforms such as WhatsApp, Instagram, or Facebook messenger have become central means of communication and have helped to create and maintain relationships. Many people perceive these apps as major platforms on which to conduct their social life [47]. According to a recent national survey [48], WhatsApp represents the most used platform among German adolescents between 12 and 19 years of age, with more than nine out of ten using it more than once per day as the main communication service. Considering that platforms such as WhatsApp are a complementary and indispensable part of everyday adolescent life, scholars should take these new forms of interactions into account when recreating the emotional experiences in the interactions with peers that could fuel the development of negative emotions that are difficult to cope with and thus increase the likelihood of adolescents’ maladjustment.

### The Present Study

The current study aims to assess a newly developed ostracism task, namely SOLO (simulated online ostracism). Due to the extensive use of instant messaging platforms such as WhatsApp as interaction and communication tools among adolescents [48], we hypothesized that SOLO would make adolescents likely experience negative affect, which will be assessed using self-reported emotional states as well as physiological reactivity (i.e., HR and HRV). This could be especially true for those adolescents who experience emotions as particularly strong and difficult to deal with, including adolescents with depression and lived experiences of self-injury. We compared the reactions to SOLO to the ones to Cyberball. Given the recent critics of the validity of Cyberball [40], we expected the self-reported emotional states as well as physiological reactivity followed by the exposure to Cyberball to be less intense compared to the ones following SOLO.

## 2. Materials and Methods

### 2.1. Participants

A total of 37 participants were recruited for this study. Due to a malfunctioning of the device used to collect data or to the dissociation of a participant, two participants were excluded from the final analyses. Thus, the analytical sample constituted a total of 35 participants (Mage = 15.16; SD = 1.48; 24 females). Of those, the majority (n = 23) reported different clinical diagnoses, all linked with an inability to cope with negative emotions, including at least five episodes of self-injury in the last year, depression, eating problems, and social phobia. The rest of the participants (n = 12) did not report any clinical diagnosis. For easier interpretability of the results, we created two different groups: a transdiagnostic group reporting emotional dysregulation, which included adolescents with a clinical diagnosis, and a control group, which included the rest of the adolescents. The participants belonging to the transdiagnostic group were patients recruited at the inpatient and outpatient unit of the clinic for children and adolescent psychiatry, psychotherapy, and psychosomatic therapy in Göppingen, Germany. The participants belonging to the control group were recruited in the German districts of Bavaria and Baden-Württemberg. To participate in the study, the adolescents needed to be between 12 and 18 years of age and needed to speak fluent German. Prior to the data collection, the study was approved by the Institutional Review Board of Ulm University, Ulm, Germany, as well as by the state medical association of Baden-Württemberg, Germany. Interested participants contacted the main author after reading the flyers promoting the study or after receiving an introduction to the study at the clinic where they were treated. Written informed consent was obtained from the participants as well as their legal guardians. Participants received a voucher for EUR 20 for participation.

Initially, we planned to recruit a balanced sample between the transdiagnostic group and the control group. However, due to entrance restrictions to the clinic where the laboratory was located, the recruitment of the control group was rendered unfeasible during the COVID-19 pandemic. While the study could be continued with inpatients (i.e., most of our transdiagnostic sample), the control group had to be limited to 12 adolescents. Nonetheless, we conducted a post hoc power analysis simulation. Our power calculation was based on a meta-analysis of 120 Cyberball studies that, on average, reported a large negative effect of the paradigm on psychological outcomes [34]. Therefore, we simulated the power to find a large effect in the chat or game condition using the R package powerlmm [49]. We based this simulation on the intra-class correlations between our four outcomes (i.e., heart rate, heart rate variability, negative and positive affect; ICC = 0.76) and our final sample size (n = 23 emotion dysregulation group, n = 12 control group). The simulation showed that our sample size still had 97% power to detect a large effect size of d = 0.8.

### 2.2. Measures

**Figure 1:**
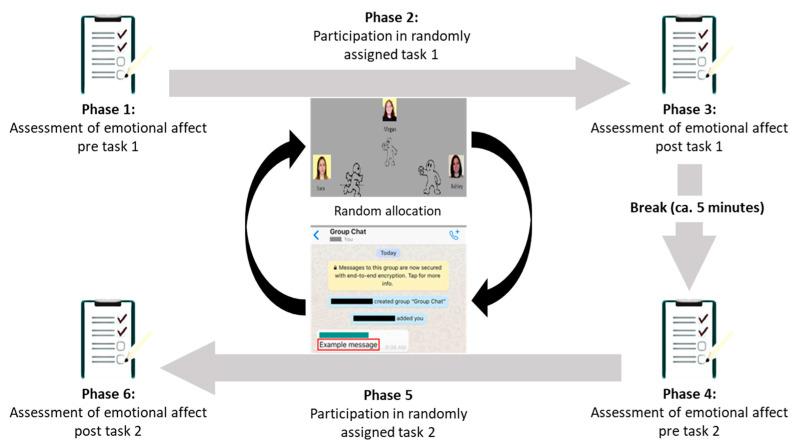
Example time plan of the experimental procedure.

In order to elicit the negative emotions linked to rejection from peers, we used the well-established Cyberball task [30], as well as our new SOLO task. To ensure a lack of biases, the order in which the tasks were presented was randomized. Information about the real nature of the study and an explanation for the necessity of deception was given after the experiment in a debriefing session. An exemplary overview of the study design is provided in Figure 1.

#### 2.2.1. Experimental Task Cyberball

Participants were made to believe that they were playing a virtual ball-tossing game with two other adolescents, currently patients of the clinic where the experiment took place. In reality, the other players did not exist, and all actions were pre-programmed [30,50]. To make the situation more realistic, we uploaded a picture of every participant below the participant’s player in the game. The pictures of the two fake participants were obtained online. All participants played two rounds of Cyberball for a total of around six minutes. In the first round (“Inclusion”; around 3 min), participants were included in the game; thus, the three players tossed the ball to each other. In the second round (“Exclusion”; around two minutes), the participant was excluded from the game, and only the two fake participants kept tossing the ball and playing with each other.

#### 2.2.2. Experimental Task SOLO

Before initiating the task, participants were told either (1) that two other participants would be participating in the same study and would have appreciated some information from our current participant on what the study entailed; or (2) that two participants already took part in the study before our current participant, and that they wanted to share some information regarding the features of the study with them. Once given these premises, the research assistant provided the participant with a cell phone where a WhatsApp chat was already organized. The chat included only the current participant and the other two participants. In reality, the other two participants were impersonated by one research assistant. The entire task consisted of two sections for a total of around 10 min. In the first section (around 5 min), the participant was included in the conversation. All three participants were writing to each other, starting to get to know each other, and sharing some information regarding the project. In the second section, the two fake participants started to ostracize the actual participants, using name-calling and mobbing. At the end of the conversation, the two fake participants agreed to take the chat somewhere else without the actual participant. Except for the different answers provided by the adolescents, the chat was standardized for all the participants. This script was based on a previous study assessing the situations over instant messaging communication platforms that are experienced as stressful by adolescents with different clinical diagnoses, including depression and non-suicidal self-injury, as well as healthy adolescents [51].

#### 2.2.3. Physiological Arousal

To assess the physiological arousal as reactions to SOLO and Cyberball, we used heart rate (HR) and heart rate variability (HRV). We recorded these indexes five minutes before and five minutes after the task. To record and collect data, we made use of an EcgMove 3 sensor (movisens GmbH, Karlsruhe, Germany) attached to the participant’s chest at the base of the sternum. We used an elastic chest belt with two integrated electrodes. All participants were sitting on a chair during the recordings. Data were visually inspected after every recording using the unisens viewer (version: 2.0). The DataAnalyzer^®^ software (movisens GmbH, Karlsruhe, Germany) was used to convert the ECG signals into time-series HR (bpm) and the RMSSD (ms). Continuous five-minute segments were analyzed for both HR and HRV.

#### 2.2.4. Negative and Positive Emotional Affect

Right before and right after each experimental task, participants filled out self-reported information regarding their current emotions. Specifically, we asked adolescents how much, on a scale from “very little” (0) to “very much” (4), they felt angry, tense, happy, and content. Using these items, we compiled a scale of negative affect (mean value between the angry and tense items) and a scale of positive affect (mean value between the happy and content items). Cronbach’s Alpha for the negative affect scale across the paradigms was 0.80, and for the positive affect scale, Cronbach’s Alpha was 0.91.

### 2.3. Statistical Analyses

All statistical analyses were conducted using R version 4.1.3. To analyze our hypotheses, we computed multilevel models with repeated measures data at level 1 (i.e., outcomes before, during, and after either paradigm) that were nested in participants. The task (SOLO or Cyberball) was modeled as a level 2 fixed effect, and the interaction task x time was tested to identify potential differences between tasks at time points.

## 3. Results

### 3.1. Descriptive Statistics

The majority of the adolescents belonging to the transdiagnostic group was constituted by females, while the control group was more homogeneous in terms of sex distribution. Slightly more than half of the transdiagnostic group reported a diagnosis of depression and/or self-injury. Significantly more adolescents belonging to the transdiagnostic group reported experiences of peer victimization, while significantly more adolescents belonging to the control group reported more experience of active aggression toward peers. More information regarding the participants can be found in Table 1.

### 3.2. Physiological Arousal

**Figure 2:**
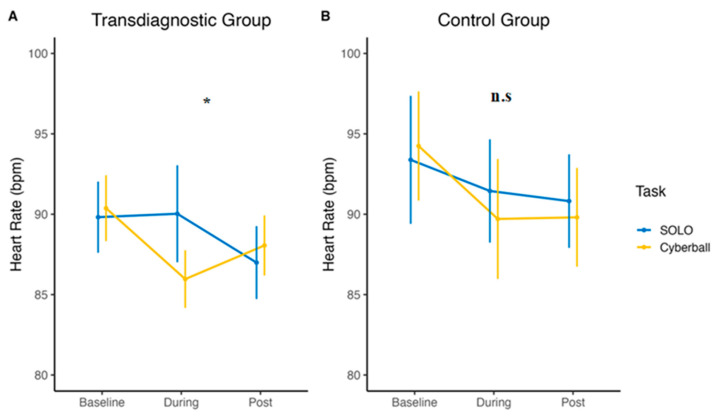
Mean HR of the transdiagnostic group (**A**) and the control group (**B**) over the two tasks. * *p* < 0.05, n.s. = non-significant.

**Figure 3:**
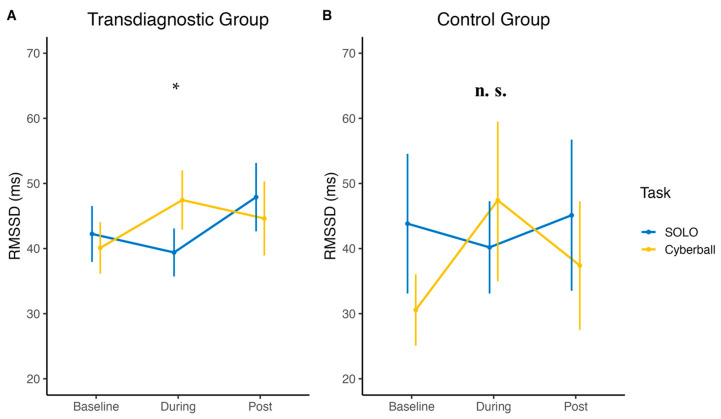
Mean HRV in the transdiagnostic group (**A**) and the control group (**B**) over the two tasks. * *p* < 0.01, n.s. = non-significant.

Adolescents in the transdiagnostic group reported higher HR during SOLO than during Cyberball (b = 4.62, *p* = 0.036, d = 0.35). In the control group, there was no difference in the effect of the task on HR (*p* = 0.335, see Figure 2). Similar to the HR results, the adolescents belonging to the transdiagnostic group showed lower HRV during the SOLO task than during Cyberball (b = 10.20, *p* = 0.008, d = −0.42). In the control group, no differences in HRV between SOLO and Cyberball were found (*p* = 0.078). See Figure 3 for a graphical representation.

### 3.3. Negative and Positive Affect

**Figure 4:**
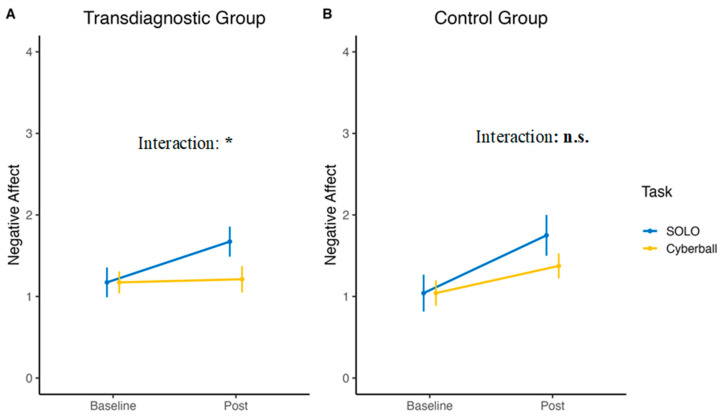
Mean negative affect of the transdiagnostic group (**A**) and the control group (**B**) over the two tasks. * *p* < 0.001, n.s. = non-significant.

When assessed, negative affect was significantly higher after SOLO than Cyberball in the transdiagnostic group (interaction: b = −0.46, *p* = 0.003, d = 0.52). In the control group, there was no difference between the negative affective response to either task (*p* = 0.827). Only an effect of time, irrespective of the task, could be identified (b = 0.44, *p* = 0.178, d = 0.94; see Figure 4).

**Figure 5:**
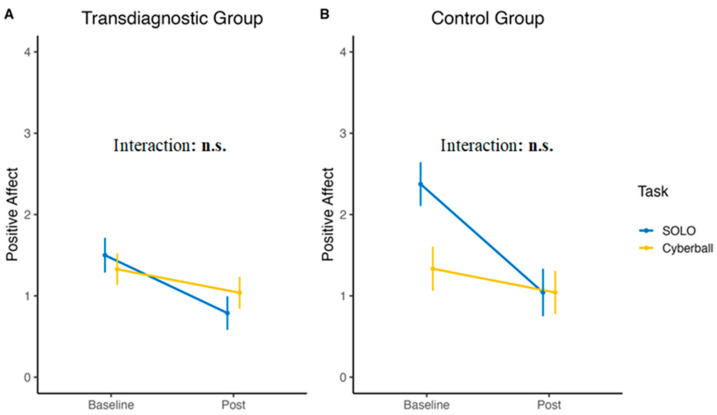
Mean positive affect of the transdiagnostic group (**A**) and the control group (**B**) over the two tasks. n.s. = non-significant.

Contrary to negative affect, positive affect responses did not differ between SOLO and Cyberball when measured in the transdiagnostic group (interaction: *p* = 0.279) or the control group (*p* = 0.080). We only found an effect of time on both the transdiagnostic (b = −0.71, *p* < 0.001, d = 0.53) and the control group (b = −1.33, *p* < 0.001, d = 0.95). See Figure 5 for a depiction of the results.

## 4. Discussion

This is the first study that assesses the psychophysiological effect of a newly developed ostracism task in laboratory settings, namely SOLO (simulated online ostracism). We proposed SOLO as an alternative to the well-known but recently criticized Cyberball task [40]. Our study showed that the transdiagnostic group of adolescents with emotion dysregulation (e.g., adolescents with depression, non-suicidal self-injury) showed higher self-reported negative affect after SOLO compared to Cyberball. They also showed higher physiological arousal (i.e., higher HR and lower HRV) during SOLO compared to the traditional Cyberball. No differences in any psychophysiological variables and between tasks were found among community adolescents who had no pre-existing clinical diagnosis.

When asked about their emotions right after both tasks, adolescents belonging to the transdiagnostic group reported an increase in negative affect (i.e., anger and tension) after SOLO. This was not the case for the adolescents belonging to the control group, who did not report any difference in negative affect after the task. These results are in line with the studies reporting high negative affectivity in adolescents with specific psychosomatic disorders, including depression [52,53,54] and self-injury [55,56]. Studies showed that adolescents who report depressive symptoms and self-injury experience high emotional reactivity [57,58,59]. These two diagnoses were met by most of the adolescents in the group. Both the cognitive model of depression [15,16] as well as the various functional models of self-injury [17,18,19] highlight the inability of these adolescents to regulate their negative emotions in an adaptive manner and when exposed to social stressors, such as ostracism.

Contrary to the negative affect, we were not able to find a decrease in positive affect (i.e., happiness, content) in the transdiagnostic group after SOLO. Researchers have shown a link between depression and low positive affect among adolescents [60], as well as a link between low positive affect and exposure to stressful events in adolescents with depression [61]. In addition, patients with depression are particularly prone to maladaptive mood repair [62,63], a maladaptive regulatory response used to attenuate sadness. The connection between low positive affect, self-injury, and inability to regulate negative emotions has been confirmed in adolescents with lived experiences [64,65,66]. Although these findings appear in contradiction with the ones presented in our study, two possible explanations could help us justify our results. First, it is possible that SOLO was either not stressful enough or not long enough to provoke the desired strong effects on changes in positive affectivity. Second, adolescents reported their positive emotions after and not during the task. Considering that during the participation in the study, all patients were undergoing psychotherapy treatment, it can be the case that they have learned how to regulate their negative emotions in an adaptive manner. For example, they could have learned reappraisal and therefore acquired the capacity to reframe the meaning of a stressful situation to alter its emotional impact. Based on these speculations, future studies should replicate our results.

When looking at the adolescents belonging to the transdiagnostic group, our results showed that their self-reported negative affect in reaction to SOLO was also present at the physiological level. Specifically, these adolescents reported an increase in HR and a decrease in HRV during the task. Lower HRV has been reported in conditions of deficient emotional regulation, such as depression [67,68]. In line with the cognitive model of depression [15,16], adolescents suffering from this diagnosis are less capable of regulating their response to negative stimuli, which can then be reflected in an alteration of the HRV. The neurovisceral integration model posits that HRV is not only an index of healthy heart function but may serve as a link between emotional regulation and healthy physiological processes [69]. In addition, other studies showed that adolescents with low HRV report an inability to inhibit impulsive behaviors in response to daily negative emotions [29]. Considering that self-injury is often performed quickly in response to negative emotions and without prior planning [18,70], this behavior perfectly fits the definition of “impulsive”. Overall, these findings could explain why only the adolescents belonging to the transdiagnostic group had a stronger reaction to SOLO.

During Cyberball, the participants belonging to both groups showed no changes in either self-reported affect or physiological arousal. In addition, there seems to be a trend showing a decrease in HR and an increase in HRV from the baseline to the execution of the task. This seems to be in line with some studies, which did not find any reliable physiological or hormonal stress response during the task [22,23]. Together with the recent criticism of the validity of Cyberball [40], these findings indicate that maybe the rejection experienced during this task may not be strong enough to elicit a full-blown stress response. The limited social interactions that participants experience during traditional Cyberball might be too artificial, too experiment-like. Furthermore, it might even decrease the level of tension that participants had at the beginning of the task and due to the novelty of the setting. This is not the case for SOLO. The WhatsApp-like format used for this new task is more ecological. As WhatsApp has become an extremely popular medium for communication [71], adolescents use it in every setting in which they interact (i.e., at home, at school, with peers, and during their leisure time). By focusing on the aspect of social interactions, SOLO was able to create the illusion of real communication, making the experimental task ecologically valid and, therefore, more triggering.

### Limitations

We should consider some limitations when discussing and interpreting the results of this study. First, due to the small sample size, as well as the differences in group sizes of the current study, the results of this study might not be generalized to the entire population. However, our results can be considered reliable when considering that the post hoc power analysis simulation showed that our sample size could detect a large effect size with 97% power. Second, the majority of the participants in the study were females. Adolescent girls are more sensitive to environmental stressors, such as ostracism and hostile interactions [72]. Thus, they may perceive these experiences as more stressful than their male counterpart. In addition, recent literature has reported valuable insights into the association between some of the diagnoses linked to emotional dysregulation (i.e., self-injury and depression) [73,74] and identifying outside the gender binary. Unfortunately, our measure does not allow us to assess adolescents who do not fit into the category of “male” or “female”. Future studies should replicate our findings, including adolescents who identify themselves as male or outside of the gender binary.

## 5. Conclusions

In the current study, adolescents with a diagnosis linked to emotional regulation problems, including depression and self-injury, responded more strongly to the newly developed SOLO task compared to the golden-standard Cyberball. Not only did they report more negative affect after the task, but they also showed stronger physiological activation (i.e., higher HR and lower HRV) during the task. Due to these findings, SOLO could be a valid and ecological alternative to Cyberball when assessing reactions to ostracism in adolescents with emotional dysregulation problems.

## Figures and Tables

**Table 1:** Descriptive information of the sample.

	Emotion Dysregulation Group (*n* = 23)	Control Group (*n* = 12)
Sex		
Females	*n* = 17	*n* = 5
Males	*n* = 6	*n* = 7
Age	Mage = 14.74 (SD = 1.389)	Mage = 16 (SD = 1.477)
Diagnosis		
Non-Suicidal Self-Injury	*n* = 13	*n* = 0
Depression	*n* = 12	*n* = 0
Social Phobia	*n* = 3	*n* = 0
Eating Disorders	*n* = 2	*n* = 0
Trauma	*n* = 1	*n* = 0
Paranoid Schizophrenia	*n* = 1	*n* = 0
Emotional disorders	*n* = 1	*n* = 0
Multiple Diagnoses	*n* = 10	*n* = 0
Aggression towards Peers	M = 1.04 (SD = 0.209) ^1^	1.43 (SD = 0.607) ^1^
Victimization from Peers	M = 1.72 (SD = 0.816) ^1^	M = 1.37 (SD = 0.168) ^1^

^1^ Likert scale ranging from 1 “It never happened” to 5 “It happened more than five times per week”.
